# Burden, trends, and predictions of five major musculoskeletal disorders in China, Japan, and South Korea: analysis based on the Global Burden of Disease Study 2021

**DOI:** 10.3389/fpubh.2025.1582618

**Published:** 2025-06-13

**Authors:** Xinyue Yin, Tingting Deng, Rui Ma, Xiaomei Fu, Ying Yin, Xuemin Jia, Fanjie Liu

**Affiliations:** ^1^Bone Biomechanics Engineering Laboratory of Shandong Province, Shandong Medicinal Biotechnology Center (School of Biomedical Sciences), Neck-Shoulder and Lumbocrural Pain Hospital of Shandong First Medical University, Shandong First Medical University & Shandong Academy of Medical Sciences, Jinan, Shandong, China; ^2^College of Traditional Chinese Medicine, Shandong University of Traditional Chinese Medicine, Jinan, China; ^3^Medical Department of Cervical, Shoulder, Neck-Shoulder and Lumbocrural Pain Hospital of Shandong First Medical University, Jinan, China; ^4^Nursing Department of Cervical, Neck-Shoulder and Lumbocrural Pain Hospital of Shandong First Medical University, Jinan, China; ^5^Acupuncture and Moxibustion Department, Affiliated Hospital of Shandong University of Traditional Chinese Medicine, Jinan, China

**Keywords:** age-period-cohort analysis, ARIMA model, rheumatoid arthritis, osteoarthritis, disease burden, joinpoint regression, high BMI

## Abstract

**Background:**

Musculoskeletal disorders impose a heavy burden on society and the healthcare system. This study aims to describe the temporal trends of the burden of the top five musculoskeletal disorders in China, Japan and South Korea, assess their age, period and cohort effects, and predict the disease burden over the next 15 years.

**Methods:**

The study utilized the Global Burden of Disease Study 2021 data. Key result metrics include the incidence, prevalence and disability-adjusted life years (DALYs) of gout, osteoarthritis, low back pain, neck pain and rheumatoid arthritis. Applying Joinpoint regression and age-period-cohort (APC) models to analyze temporal trends of five major musculoskeletal diseases from 1990 to 2021, considering age, period, and cohort effects. Future burden from 2022 to 2036 was forecasted using the autoregressive integrated moving average (ARIMA) model.

**Results:**

From 1990 to 2021, the incidence of gout and osteoarthritis in China increased by 160.5 and 150.4%, respectively, with Japan’s gout incidence up by 65.3% and South Korea’s gout and rheumatoid arthritis rates rising by 165.0 and 164.6%. The burden of these diseases, except for gout, was higher in female. The risk of musculoskeletal diseases increases with age and recent birth cohorts. The impact of high body mass index (BMI) on gout, low back pain, and osteoarthritis is escalating. By 2036, the disease burden is expected to remain heavy, with varying degrees of increase.

**Conclusion:**

The burden of the five musculoskeletal diseases in China, Japan, and South Korea is significant. There is a particular need to pay close attention to the increasingly severe impact of older adult female populations and high BMI on gout, low back pain, and osteoarthritis. It is recommended that governments increase investment and implement prevention strategies, including weight management and health education.

## Highlights

1. First comprehensive analysis of five major musculoskeletal disorders in China, Japan, and South Korea using GBD 2021 data, revealing significant increases in gout (up 165% in South Korea) and osteoarthritis (150% in China) from 1990 to 2021.
2. Rising BMI identified as a critical driver of disease burden, particularly for gout, low back pain, and osteoarthritis, with older adult female disproportionately affected, underscoring the need for targeted weight management and health education.
3. Projections indicate persistent disease burden over the next 15 years, with gout in Japan/South Korea and low back pain in China expected to rise, urging policymakers to prioritize aging populations and obesity-related interventions.

## 1 Introduction

The global trend of population aging is intensifying day by day, and many countries are facing challenges brought about by changes in population structure. China, Japan, and South Korea, as representative countries in East Asia, were all confronted with the severe problem of population aging in recent years. Among them, Japan is one of the countries with the most severe population aging in the world, with an aging rate (the proportion of the population aged 65 and above in the total population) reaching 29.1%. In addition, the aging rates in South Korea and China have also been increasing year by year, approximately 19.2 and 13.5%, respectively (1). The negative impacts brought about by population aging are also intensifying. As people age, the degenerative changes in the human musculoskeletal system become increasingly obvious, leading to an increase in the prevalence of musculoskeletal diseases among the older adult. Moreover, in 2020, musculoskeletal disorders ranked as the second most significant contributor to non-fatal disabilities globally, impacting more than 16.3 billion individuals (2–4). These conditions have placed substantial financial and healthcare pressures on individuals, families, and governmental systems. In an early Global Burden of Disease (GBD) study in 2010, musculoskeletal diseases were the fourth leading factor affecting global population health (5). It is estimated that approximately 6.7% of disability-adjusted life years (DALYs) worldwide are attributed to musculoskeletal diseases (6, 7). When measured by disability life years, 21% of global disability is attributed to musculoskeletal diseases (8, 9). With the extension of global life expectancy (10), more efforts are needed to maximize healthy life expectancy, that is, the additional life gained is spent in a healthy state (11). According to epidemiological studies, the top five main diseases that cause the greatest burden on musculoskeletal systems include gout, low back pain, neck pain, osteoarthritis, and rheumatoid arthritis.

Although numerous previous studies have reported the global burden of musculoskeletal-related diseases, these studies have limitations: some studies only focus on a specific disease, such as gout (12) and osteoarthritis (13), ignoring the intrinsic correlations among musculoskeletal diseases; some studies use data mostly from the GBD 2019 or 2017 versions, which have poor data timeliness and limited sample coverage (14–17). The new data from the 2021 GBD study was recently released, and there is currently no comprehensive analysis of the five major musculoskeletal diseases in China, Japan, and South Korea based on these data. Therefore, we conducted a study on the burden of the five major musculoskeletal diseases in the three countries from 1990 to 2021, including analyzing their temporal trends, assessing their risk factors, and evaluating the effects of age, period, and cohort. In addition, we used the autoregressive integrated moving average (ARIMA) model to predict the burden over the next 15 years. Our analysis results will play a key role in providing information for decision-makers and the public, helping to formulate evidence-based public policies, and effectively allocating prevention resources.

## 2 Materials and methods

### 2.1 Data source

Data on the burden of the five primary musculoskeletal disorders in China, Japan, and South Korea from 1990 to 2021 was obtained from the GBD 2021 Public Database^1^. The GBD 2021 analysis of disease and injury burden used 100,983 data sources and applied the Bayesian meta-regression model DisMod-MR 2.1 to estimate mortality, incidence, prevalence, and associated disability rates for 371 diseases, injuries, and impairments, as well as 88 risk factors, stratified by year, age, and sex. This comprehensive study included 204 nations and territories, leveraging the most current epidemiological information and enhanced standardization techniques (18, 19).

### 2.2 Case definition

The International Classification of Diseases, Ninth Revision (ICD-9) and the Tenth Revision (ICD-10) codes were, respectively, used to define rheumatoid arthritis (ICD-9: 714.0–714.9; ICD-10: M05, M06, M08), osteoarthritis (ICD-9: 715; ICD-10: M16, M17), low back pain (ICD-9: 724; ICD-10: M54.3, M54.4, M54.5), neck pain (ICD-9: 723.1; ICD-10: M54.2) and gout (ICD-9: 274; ICD-10: M10).

### 2.3 Risk factors

In the GBD 2021 study, the risk factors associated with the five primary musculoskeletal conditions were delineated. Rheumatoid arthritis was linked to smoking, while osteoarthritis was associated with a high BMI. The factors contributing to low back pain included smoking, high BMI, and occupational ergonomic factors. Gout was attributed to both a high BMI and impaired kidney function. High BMI for adults (ages 20 and older) is defined as BMI greater than 20–23 kg/m^2^. High BMI for children (ages 2–19) is defined as being overweight or obese based on International Obesity Task Force standards (20).

### 2.4 Joinpoint regression model

Kim et al. (21) firstly proposed the Joinpoint regression model in 2000. This approach built segmented regressions by utilizing the temporal traits of disease distribution, executing trend fitting and refinement for individual data points within the series. It facilitated a comprehensive analysis of the distinct characteristics of disease variability across various global intervals. The formula for the log-linear model was $\;E[y|x]=e^{\beta_{0}+\beta_{1}x+\delta_{1}{\left(x-\tau_{1}\right)}^{+}+\ldots +\delta_{k}{\left(x-\tau_{k}\right)}^{+}}$ , y denoted the prevalence or occurrence of the disease, x corresponded to the year, β_1_ was the coefficient of regression, k denoted the joinpoint’s index, τ_k_ was the joinpoint whose value was unknown, “a^+^ = a” indicated that the result was greater than 0, otherwise, it was 0. Outcomes of the model can be encapsulated with metrics such as the annual percentage change (APC) and the average age annual percentage change (AAPC). The APC was computed as $\mathrm{APC}=[\frac{y_{x+1}-y_{x}}{y_{x}}]*100\%=(e^{\beta_{1}}-1)*100\%$ , which evaluated the trend within the separate segments of the piecewise function. The formula for calculating the AAPC was $\text{AAPC}=(e^{\sum \frac{\omega_{i}\beta_{i}}{\sum \omega_{i}}}-1)*100\%$ , providing an assessment of the overall trend across the complete study period.

### 2.5 Age-period-cohort model

The Age-Period-Cohort (APC) Model investigated how age, period, and cohort factors influenced health outcomes. The age effect pertained to the varying risk of outcomes at distinct age levels. The period effect concerned the influence of time-related shifts on outcomes across all age categories. The cohort effect related to the differences in outcomes among individuals born in the same time period. The log-linear regression equation was $\log(Y_{i})=\mu +\alpha *{\mathrm{age}}_{i}+\beta *{\text{period}}_{i}+\gamma *{\text{cohort}}_{i}+\varepsilon$ , $Y_{i}$ denoted the incidence or death rate, α, β, and γ were the parameters for age, period, and cohort, respectively, μ was the constant term, and ε denoted the error term of the model. The Intrinsic Estimator technique, incorporated within the Age-Period-Cohort Model, was applied to isolate the pure effects within the three-dimensional framework (22).

### 2.6 Autoregressive integrated moving average (ARIMA) model

The Autoregressive Integrated Moving Average (ARIMA) model was an amalgamation of the Autoregressive (AR) model and the Moving Average (MA) model. It operated on the premise that the data set behaves as a stochastic process, and its variability can be modeled by ARIMA, enabling predictions of future data points based on historical observations. The mathematical representation was given by the following equation, $Y_{t}=\varphi_{1}Y_{t-1}+\varphi_{2}Y_{t-2}+\ldots +\varphi_{p}Y_{t-p}+e_{t}-\theta_{1}e_{t-1}-\ldots -\theta_{q}e_{t-q},$ where denoted the AR component was $\left(\varphi_{1}Y_{t-1}+\varphi_{2}Y_{t-2}+\ldots +\varphi_{p}Y_{t-p}+e_{t}\right)$ , $e_{t}-\theta_{1}e_{t-1}-\ldots -\theta_{q}e_{t-q}$ signified the MA component, Y_t − p_ was the observed value for the period (t − p), p and q were the order parameters for the AR and MA models, respectively, and e_t_ was the error term at time t during the period (23). For the ARIMA model, the time series data was required to be a stationary process with a mean of zero.

### 2.7 Statistical analysis

For the GBD 2021 study, the age-standardized incidence rate (ASIR) and age-standardized DALYs rate (ASDR) were computed with the global population serving as the standard reference. The Joinpoint Regression Program (version 5.2.0) was employed for the analytical purposes. To conduct the age-period-cohort analysis, the dataset was segmented into five continuous intervals spanning from 1992 to 2021. Data for the years 1990 to 1992 were excluded due to their failure to encompass complete five-year intervals. The age groups were also divided into five-year increments, starting from 15 to 19 years and extending to 95–99 years. Individuals below the age of 15 were not included in the age-period-cohort analysis (with those under 25 years excluded for osteoarthritis). Furthermore, the analysis summarized 20 cohorts, encompassing individuals born between 1905–1909 and 2000–2004, with the mean values of age, period, and cohort serving as the reference category (24, 25). During the ARIMA modeling phase, the differencing technique was initially applied to achieve stationarity in the time series data. The auto.arima() function was utilized to identify the most optimally fitted model according to the Akaike Information Criterion (AIC) (26). Q–Q plots, along with Autocorrelation Function (ACF) and Partial Autocorrelation Function (PACF) plots, were employed to assess the normality of the residuals’ distribution. Subsequently, the Ljung-Box test was conducted to determine if the residual series constituted white noise. ARIMA analysis and graphical representation were carried out using the “forecast,” “tseries,” and “ggplot2” packages within the R 4.4.2 software, with a significance level of *p* < 0.05.

## 3 Results

### 3.1 Cross-sectional analysis

From 1990 to 2021, the five major musculoskeletal diseases were rheumatoid arthritis, osteoarthritis, low back pain, neck pain and gout. Gout had seen the greatest change in disease burden over the past three decades. In China, the incidence and DALYs of the five major musculoskeletal diseases had all been on the rise (Table 1). In terms of incidence, the percentage changes that were the greatest were gout (160.5% [95% UI 140.1–178.5%]) and osteoarthritis (150.4% [95% UI 142.8–157.1%]). In terms of DALYs, the percentage changes that were the greatest compared to 1990 were also osteoarthritis (191.2% [95% UI 183.6–198.7%]) and gout (176.0% [95% UI 157.0–195.7%]). The incidence and DALYs of the five diseases in South Korea were similar to those in China, with significant increases. Among them, gout had the largest percentage change in the number of cases and DALYs, at 165.0% [95% UI 138.4–191.5%] and 205.3% [95% UI 163.7–255.5%], respectively. In Japan, the burden growth was slightly lower than that of China and South Korea, and the highest percentage increase in the number of cases was also for gout (65.3% [95% UI 51.4–82.2%]). The other four diseases showed relatively smaller increases, but still maintained a positive growth trend (Table 1).

**Table 1:** Numbers of incidence and DALYs from five major musculoskeletal disorders in China, Japan and South Korea in 1990 and 2021, and the percentage changes from 1990 to 2021.

Location (causes)	Incidence (95% CI)			DALYs (95% CI)		
	1990	2021	Percentage change, 1990–2021 (%)	1990	2021	Percentage change, 1990–2021 (%)
China						
Rheumatoid arthritis	127825.8 (111476.8–145914.4)	247307.0 (216204.8–282997.6)	93.5 (79.7–109.3)	403058.4 (307641.2–526243.7)	833817.9 (621520.2–1083523.5)	106.9 (86.1–125.6)
Osteoarthritis	4654141.5 (4075192.0–5212885.6)	11652721.2 (10207638.2–13107929.2)	150.4 (142.8–157.1)	1829415.6 (880107.8–3682519.9)	53273890.0 (2541781.2–10678674.9)	191.2 (183.6–198.7)
Low back pain	29843969.7 (26065823.6–34012369.4)	43374995.0 (37494375.5–49159184.3)	45.3 (37.6–53.1)	7772957.8 (5520145.0–10545675.6)	11297804.9 (7931467.8–15328056.4)	45.3 (38.8–53.2)
Neck pain	6192332.2 (4778922.4–7748853.2)	10292099.2 (8062750.7–13039595.5)	66.2 (44.8–87.4)	2675172.1 (1740002.2–3911198.5)	4807593.4 (3155339.5–6903309.6)	79.7 (58.7–103.2)
Gout	1182498.0 (940489.8–1461668.8)	3079835.5 (2425497.6–3891397.7)	160.5 (140.1–178.5)	190614.2 (126028.0–276352.5)	525967.2 (353075.3–758228.8)	176.0 (157.0–195.7)
Japan						
Rheumatoid arthritis	31521.2 (27205.8–36783.2)	32060.1 (27599.3–37007.6)	1.7 (−4.6–8.3)	100774.5 (79027.3–129604.7)	122653.9 (93498.6–158301.1)	21.7 (17.1–26.1)
Osteoarthritis	1083158.1 (956009.7–1200740.9)	1510140.7 (1348295.0–1664133.4)	39.4 (34.5–44.7)	493607.6 (237042.9–994223.8)	939485.0 (450468.5–1907691.6)	90.3 (85.2–96.1)
Low back pain	7134686.8 (6286926.2–8008476.2)	8085995.2 (7116421.3–9043617.7)	13.3 (8.4–19.2)	1974321.9 (1399797.6–2673927.3)	2232624.4 (1590995.6–2995119.9)	13.1 (8.2–18.5)
Neck pain	766822.0 (596081.1–950552.7)	863185.0 (680042.4–1062102.8)	12.6 (4.1–23.0)	366328.5 (241787.0–521848.6)	418836.9 (276061.7–588451.5)	14.3 (5.9–25.0)
Gout	176964.6 (138855.0–225138.6)	292599.8 (228340.3–374291.2)	65.3 (51.4–82.2)	34025.0 (23048.3–48870.8)	60716.4 (40826.2–88089.4)	78.4 (63.8–94.8)
Republic of Korea						
Rheumatoid arthritis	4135.2 (3688.5–4581.2)	10942.0 (9830.8–12138.8)	164.6 (139.8–189.9)	12755.9 (9835.9–16506.4)	30097.1 (21480.5–40114.1)	136.0 (104.2–168.0)
Osteoarthritis	243702.2 (215123.0–270409.5)	615472.0 (547205.3–688215.6)	152.6 (143.1–162.9)	96296.4 (46362.8–195129.9)	307676.8 (148214.6–623772.0)	219.5 (208.5–231.0)
Low back pain	1752694.2 (1528642.7–1999909.0)	2704830.9 (2366623.4–3045723.8)	54.3 (44.3–65.6)	461879.7 (326978.0–619749.3)	727107.5 (516988.9–977658.2)	57.4 (45.7–70.4)
Neck pain	166719.2 (127809.1–207427.2)	238988.7 (185048.1–305344.1)	43.3 (25.2–61.6)	82975.2 (53622.2–121167.2)	130375.9 (84832.4–186196.4)	57.1 (38.6–74.0)
Gout	34248.1 (26592.7–42612.8)	90755.4 (70548.3–115113.8)	165.0 (138.4–191.5)	6538.0 (4252.0–9773.4)	20001.6 (13384.5–29031.1)	205.3 (163.7–255.5)

Data are shown as N (95% uncertainty interval).

As people grow older, the prevalence rates of gout and osteoarthritis in China gradually increased (Supplementary Figures 1A,G), while the incidence rates of low back pain, neck pain and rheumatoid arthritis first rose and then fell, with the peaks concentrated between 65 and 90 years old (Supplementary Figures 1B–D). The trend of incidence rates was basically the same as that of prevalence rates, except that the incidence rate of osteoarthritis showed a difference from the prevalence rate. It increased sharply between 30 and 54 years old, and then showed an overall downward trend with age. In Japan, as people age, the incidence rate and prevalence rate of gout gradually increased, and neck pain showed a double peak, and the disease burden was at a relatively high level between 40 and 95+ years old (Supplementary Figure 1H). The peaks of the other three diseases were between 60 and 90 years old. In South Korea, the trend of incidence rate and prevalence rate was similar to that of Japan. The incidence rate and prevalence rate of neck pain were both higher in the 40–95 + age group (Supplementary Figure 1M). The peaks of the other four diseases were concentrated between 50 and 80 years old. And except for the male disease burden of gout being higher than that of females, the others were all higher in females than in males.

**Figure 1:**
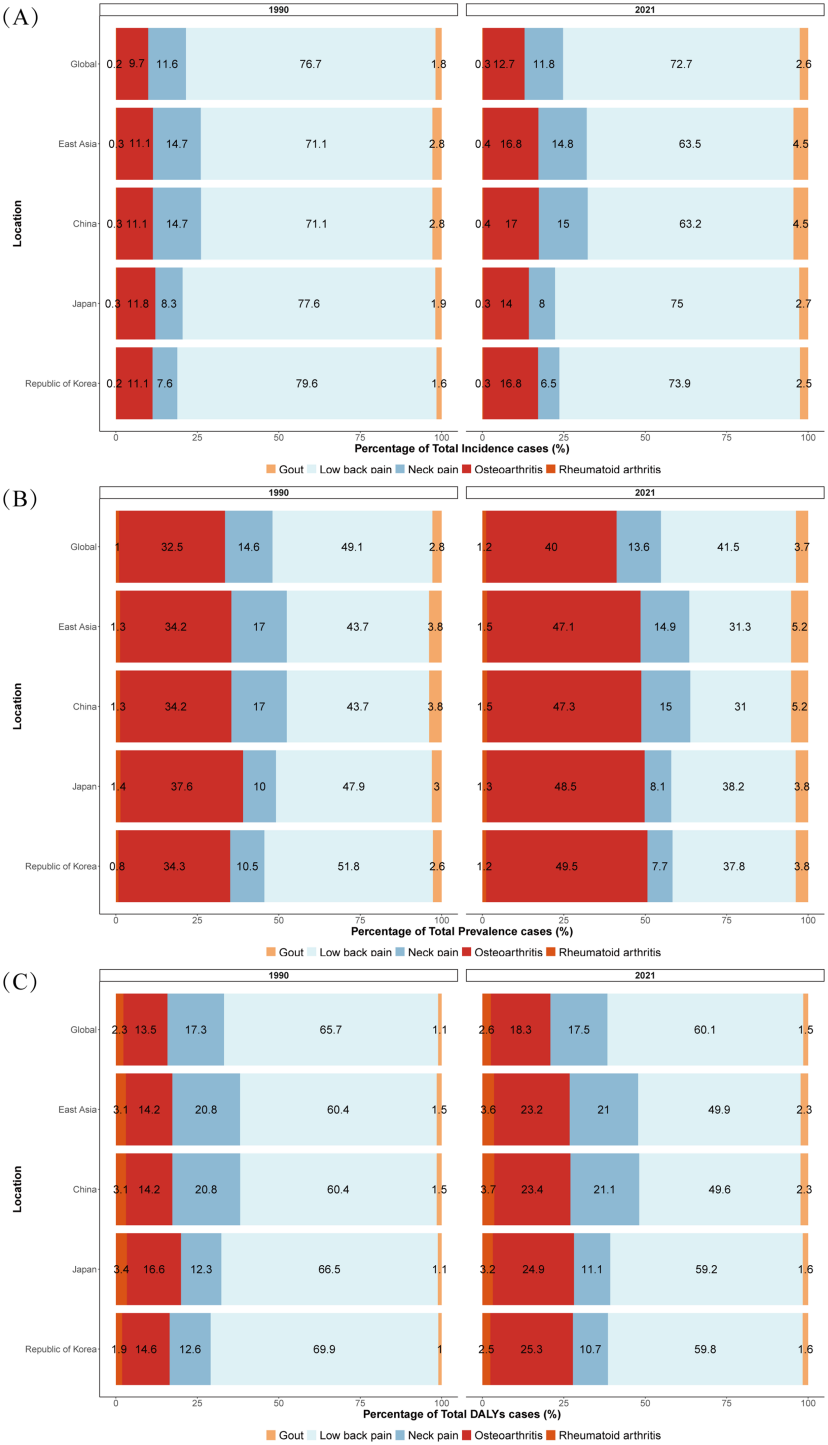
Percentage of total number of cases **(A)**, number of patients **(B)** and disability-adjusted life years **(C)** of the five major musculoskeletal diseases in the world, East Asia, China, Japan and South Korea.

From 1990 to 2021, the age-standardized incidence and prevalence rates of gout and osteoarthritis in China had increased to varying degrees. The age-standardized incidence and prevalence rates of gout slightly decreased during the period of 1990–1996, and then increased to a small extent (Supplementary Figure 2A). The age-standardized incidence and prevalence rates of osteoarthritis were the highest in 2005 and then tended to stabilize (Supplementary Figure 2D). The age-standardized incidence and prevalence rates of low back pain decreased year by year from 1990 to 1995, and then stabilized (Supplementary Figure 2B). The age-standardized incidence and prevalence rates of five musculoskeletal diseases in Japan were much more stable compared to those in China. The age-standardized incidence and prevalence rates of osteoarthritis and rheumatoid arthritis in females increased year by year from 2006 to 2010, and then decreased starting from 2015, returning to the previous levels (Supplementary Figures 2I,J). The age-standardized incidence and prevalence rates of rheumatoid arthritis among females in South Korea slightly increased (Supplementary Figure 2O). Compared with 1990, in 2021, the prevalence of osteoarthritis in the total number of patients, the total number of cases, and the disability-adjusted life years in the five major musculoskeletal diseases in China had all increased significantly. The percentages of gout and rheumatoid arthritis in the total number of patients and the total number of cases had increased to varying degrees (Figure 1).

### 3.2 Joinpoint regression analysis

**Figure 2:**
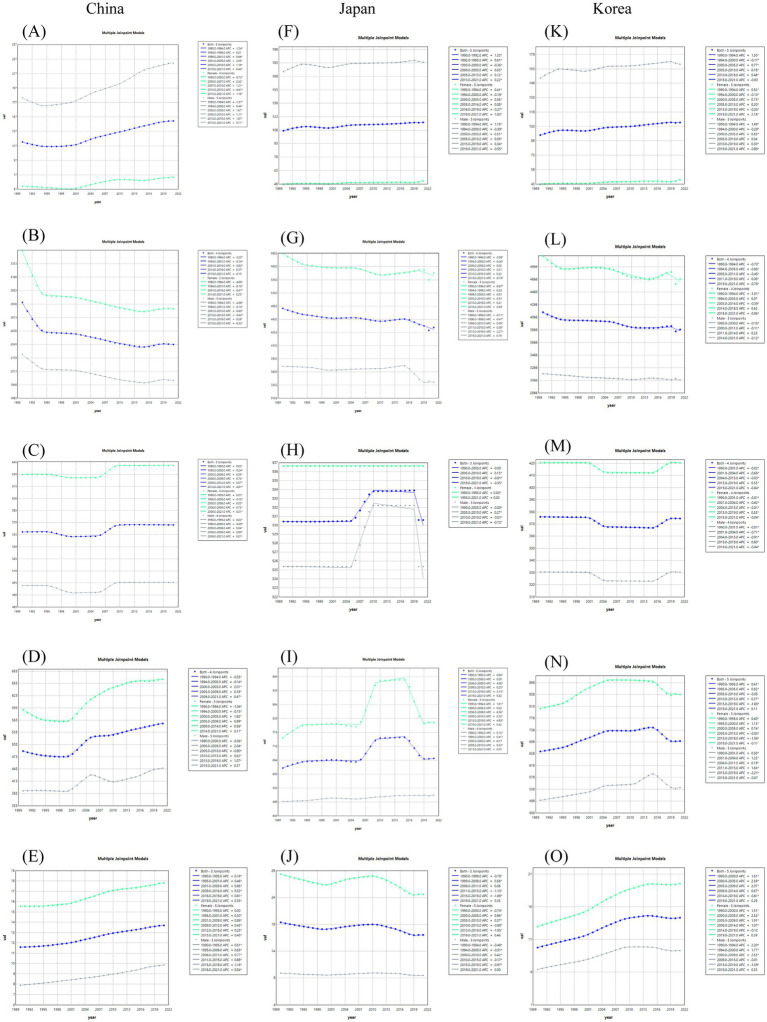
Joinpoint regression analysis of the connection points of ASIR for the five major musculoskeletal diseases in China, Japan and South Korea. Gout **(A,F,K)**, Low back pain **(B,G,L)**, Neck pain **(C,H,M)**, Osteoarthritis **(D,I,N)**, Rheumatoid arthritis **(E,J,O)**.

**Figure 3:**
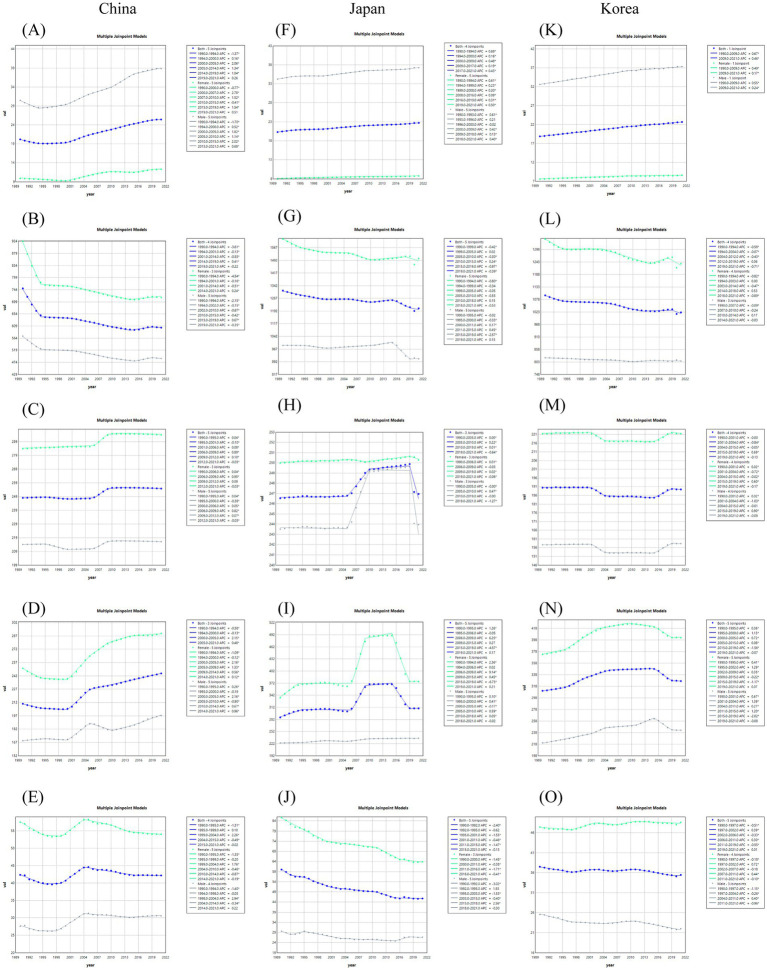
Joinpoint regression analysis of the connection points of the top five musculoskeletal diseases (ASDR) in China, Japan and South Korea. Gout **(A,F,K)**, Low back pain **(B,G,L)**, Neck pain **(C,H,M)**, Osteoarthritis **(D,I,N)**, Rheumatoid arthritis **(E,J,O)**.

The trend of segmental changes of ASIR and ASDR for the five major musculoskeletal diseases in China, Japan and South Korea was analyzed using the Joinpoint regression analysis. Since 1990, the ASIR of gout in China had increased significantly twice during the periods of 2001–2005 and 2005–2018, with APC of 2.05 and 1.18%, respectively, (Figure 2A). The ASDR had also increased significantly twice during the periods of 2000–2005 and 2005–2014, with APC of 2.06 and 1.34%, respectively, (Figure 3A). The AAPC of ASIR and ASDR throughout the period was 0.70% (95% CI 0.64–0.76%) and 0.75% (95% CI 0.71–0.80%) respectively (Table 2). The ASIR and ASDR of low back pain showed a downward trend from 1990 to 2021, with AAPC of −0.63% (95% CI -0.67% ~ −0.58%) and −0.68% (95% CI -0.73% ~ −0.64%) respectively (Table 2). The ASIR and ASDR of low back pain showed a significant decline from 1990 to 1994, with APC of −3.23% and −3.61%, respectively, (Figures 2B, 3B). The ASIR and ASDR of neck pain remained stable overall (Figures 2C, 3C). The trends of ASIR and ASDR of osteoarthritis were basically consistent over the past 30 years. They gradually decreased from 1990 to 2000, and then increased significantly once from 2000 to 2005, with APC of 2.01 and 2.05%, respectively, (Figures 2D, 3D). The ASIR of rheumatoid arthritis showed a steadily increasing trend, while the ASDR increased significantly from 1999 to 2004 with APC = 2.26% (Figures 2E, 3E).

**Table 2:** The AAPC in age-standardized rate of incidence and DALYs for five major musculoskeletal disorders in China, Japan and South Korea.

Location (causes)	Incidence (95% CI)			DALYs (95% CI)		
	Both	Female	Male	Both	Female	Male
China						
Rheumatoid arthritis	0.54 (0.53–0.56)*	0.44 (0.41–0.47)*	0.72 (0.69–0.74)*	−0.02 (−0.13–0.09)	−0.21 (−0.31 – −0.10)*	0.32 (0.16–0.47)*
Osteoarthritis	0.43 (0.39–0.46)*	0.39 (0.34–0.43)*	0.42 (0.36–0.48)*	0.49 (0.46–0.52)*	0.47 (0.42–0.52)*	0.51 (0.43–0.59)*
Low back pain	−0.63 (−0.67 – −0.58)*	−0.71 (−0.75 – −0.67)*	−0.50 (−0.53 – −0.46)*	−0.68 (−0.73 – −0.64)*	−0.79 (−0.84 – −0.75)*	−0.50 (−0.54 – −0.46)*
Neck pain	0.05 (0.05–0.05)*	0.06 (0.06–0.06)*	0.03 (0.02–0.03)*	0.09 (0.08–0.10)*	0.11 (0.10–0.13)*	0.03 (0.02–0.04)*
Gout	0.70 (0.64–0.76)*	0.61 (0.49–0.73)*	0.77 (0.69–0.84)*	0.75 (0.71–0.80)*	0.68 (0.56–0.81)*	0.81 (0.74–0.88)*
Japan						
Rheumatoid arthritis	−0.41 (−0.47 – −0.34)*	−0.44 (−0.51 – −0.38)*	−0.14 (−0.16 – −0.12)*	−0.88 (−1.04 – −0.72)*	−0.95 (−1.05 – −0.84)*	−0.35 (−0.69 – −0.01)*
Osteoarthritis	0.18 (0.05–0.31)*	0.25 (0.10–0.39)*	0.15 (0.11–0.20)*	0.23 (0.04–0.42)*	0.36 (0.12–0.59)*	0.16 (0.15–0.18)*
Low back pain	−0.28 (−0.36 – −0.19)*	−0.20 (−0.30 – −0.10)*	−0.28 (−0.33 – −0.22)*	−0.30 (−0.39 – −0.21)*	−0.24 (−0.33 – −0.15)*	−0.26 (−0.28 – −0.24)*
Neck pain	−0.00 (−0.00 – −0.00)*	0.00 (0.00–0.00)	−0.01 (−0.01 – −0.01)*	−0.00 (−0.01–0.00)	0.00 (−0.00–0.01)	−0.01 (−0.02 – −0.00)*
Gout	0.27 (0.23–0.32)*	0.23 (0.20–0.26)*	0.18 (0.15–0.22)*	0.36 (0.34–0.38)*	0.29 (0.26–0.31)*	0.27 (0.25–0.30)*
Republic of Korea						
Rheumatoid arthritis	1.23 (1.16–1.30)*	1.31 (1.26–1.37)*	1.19 (1.12–1.26)*	−0.19 (−0.24 – −0.14)*	0.07 (−0.01–0.14)	−0.54 (−0.63 – −0.45)*
Osteoarthritis	0.17 (0.14–0.19)*	0.18 (0.13–0.23)*	0.26 (0.22–0.29)*	0.17 (0.15–0.19)*	0.24 (0.19–0.30)*	0.32 (0.24–0.39)*
Low back pain	−0.23 (−0.26 – −0.20)*	−0.27 (−0.43 – − 0.10)*	−0.10 (−0.16 – −0.04)*	−0.24 (−0.28 – −0.21)*	−0.29 (−0.44 – −0.14)*	−0.06 (−0.11–0.00)
Neck pain	−0.01 (−0.02 – −0.01)*	−0.00 (−0.00 – − 0.00)*	−0.00 (−0.00 – −0.00)*	−0.02 (−0.05–0.01)	−0.00 (−0.03–0.02)	0.01 (−0.02–0.04)
Gout	0.41 (0.38–0.45)*	0.32 (0.30–0.34)*	0.28 (0.24–0.31)*	0.59 (0.58–0.61)*	0.37 (0.34–0.40)*	0.43 (0.41–0.45)*

AAPC, average annual percent changes; CI, confidence interval. *The AAPC is significantly different from zero at the alpha = 0.05 level.

The ASDR of gout in Japan had been increasing year by year over the past three decades. The ASIR showed a downward trend from 1995 to 2000, but it remained on the rise during other periods, with the largest increase occurring between 1990 and 1992, with an APC of 1.33% (Figures 2F, 3F). The overall ASIR of low back pain showed a decreasing trend, and the rate of decline has accelerated in recent years, with an APC of −0.74% from 2015 to 2021. The ASDR also showed a similar trend, with an overall decreasing trend and a significant decline from 2015 to 2019 (APC = 0.97%) (Figures 2G, 3G). The trends of ASIR and ASDR for neck pain were basically consistent. They both showed a significant decline from 2019 to 2021, with APCs of −0.35% and −0.64%, respectively, (Figures 2H, 3H). The ASIR and ASDR of osteoarthritis in Japan were also basically consistent in trend. They both showed a significant increase from 2006 to 2009, with APCs of 4.06 and 6.25%, respectively. They both showed a significant decline from 2015 to 2019, with APCs of −3.13% and −3.57%, respectively (Figures 2I, 3I). The ASIR of rheumatoid arthritis in Japan showed a decreasing trend overall, with the fastest decline from 2015 to 2019 (APC = −1.69%), and the overall APC was −0.41% (95% CI −0.47% to −0.34%). The ASDR showed a decreasing trend year by year, with the fastest decline from 1995 to 2001 (APC = −1.47%), and the overall APC was −0.88% (95% CI −1.04% to −0.72%) from 1990 to 2021 (Figures 2J, 3J and Table 2).

Over the past three decades, both the ASIR and ASDR of gout in South Korea had shown an upward trend. The ASIR increased the fastest between 1990 and 1994, with an APC of 1.30%. The AAPCs were 0.41% (0.38–0.45%) and 0.59% (0.58–0.61%), respectively (Figures 2K, 3K and Table 2). The trends of ASIR and ASDR for low back pain were essentially consistent, showing an overall decrease, with the fastest decline occurring between 2019 and 2021, with APCs of −0.78% and −0.71%, respectively (Figures 2L, 3L). The trends of ASIR and ASDR for neck pain were also largely consistent, showing a rapid decrease between 2001 and 2004, with APCs of −0.66% and −0.84%, respectively, followed by a year-on-year increase between 2015 and 2019, with APC of 0.55 and 0.69%, respectively (Figures 2M, 3M). The ASIR of osteoarthritis showed an overall upward trend but experienced a significant decrease between 2015 and 2019 (APC = −1.68%). Its ASDR increased year by year from 1990 to 2005 and then significantly decreased between 2015 and 2019 (APC = −1.56%) (Figures 2N, 3N). The ASIR of rheumatoid arthritis showed an overall upward trend, with two significant increases occurring between 2000 and 2005 and between 2005 and 2009 (APC 2000–2005 = 2.59%, APC 2005–2009 = 2.07%), resulting in an AAPC of 1.23% (95% CI 1.16–1.30%) for the entire period. Notably, the trend of ASDR was different from that of ASIR, showing an overall downward trend with significant fluctuations between 1997 and 2011 (Figures 2O, 3O and Table 2).

### 3.3 Age, period and cohort

Supplementary Figure 3 illustrates the age-period-cohort trends of the incidence rates of the five major musculoskeletal diseases in China, Japan, and South Korea. The incidence rates of musculoskeletal diseases gradually increased with age, and the peak occurred in the range of 60–80 years old. The cohort risk of gout in the recent birth cohort in China had slightly increased [RRcohort (2000–2004) = 1.47, 95% CI 1.14–1.89]. The incidence rate based on period has also increased year by year [RRperiod (2017–2021) = 1.20, 95% CI 1.18–1.22] (Supplementary Figure 3A). The cohort risk of low back pain had gradually increased in the recent birth cohort (RRcohort 1992 = 0.84, 95% CI 0.81–0.86), (RRcohort 2002 = 0.92, 95% CI 0.87–0.97) in recent years. The incidence rate had increased in the younger age group in recent years, and the onset time of low back pain had become earlier. The incidence rate based on period had decreased year by year since 1992, and the decline was the greatest from 1992 to 2016 [RRperiod (2012–2016) = 0.96, 95% CI 0.94–0.97] (Supplementary Figure 3B). The incidence rate based on period had increased the most in the range of 2017–2021 for neck pain [RRperiod (2017–2021) = 1.04, 95% CI 1.01–1.07] (Supplementary Figure 3C). The prevalence diagnosis rate of osteoarthritis was the highest around 50–60 years old, and the incidence rate based on period had gradually increased since 1997 [RRperiod (2017–2021) = 1.05, 95% CI 1.00–1.11] (Supplementary Figure 3D). The incidence rate of rheumatoid arthritis peaked around 60 years old and then decreases with age. The incidence rate based on period had gradually increased [RRperiod (2017–2021) = 1.11, 95% CI 1.06–1.16] (Supplementary Figure 3E). The risk of the birth cohort had increased from 1.29 (95% CI 1.24–1.32) in 1992 to 1.44 (95% CI 1.35–1.53) in 2002 (Supplementary Figure 3E).

The incidence of gout in Japan had been increasing year by year since 1997, with the relative risk (RR) being 0.98 (95% CI 0.98–0.99) for the period from 1997 to 2001. The risk of gout in the birth cohort rose from 0.94 (95% CI 0.90–0.97) in 1902 to 1.11 (95% CI 0.93–1.32) in 2002 (Supplementary Figure 3F). The incidence of low back pain increased significantly with age, reaching a peak around 80 years old and then slightly decreasing. The incidence based on the period decreased year by year since 1992, with the relative risk being 1.02 (95% CI 1.02–1.03) for the period from 1992 to 2006 and 0.97 (95% CI 0.97–0.98) for the period from 2017 to 2021 (Supplementary Figure 3G). The risk of low back pain decreased from 1.07 (95% CI 1.03–1.12) in 1902 to 0.92 (95% CI 0.89–0.94) in 2002 (Supplementary Figure 3H). The incidence of neck pain gradually increased with age. The incidence based on the period remained stable from 1992 to 2006 and gradually increased from 2007 to 2016, and then gradually decreased from 2017 to 2021. The risk of neck pain in the birth cohort fluctuated in recent years (Supplementary Figure 3H). The incidence of osteoarthritis based on the period increased from 1992 to 1996 and from 2002 to 2016, and decreased from 1997 to 2001 and from 2017 to 2021. The risk of osteoarthritis in the birth cohort rose from 1.18 (95% CI 1.01–1.29) in 1992 to 1.23 (95% CI 0.91–1.74) in 2002 (Supplementary Figure 3I). The incidence of rheumatoid arthritis increased significantly with age and reached a peak between 60 and 80 years old. The incidence based on the period fluctuated slightly but showed a downward trend, with the relative risk being 1.02 (95% CI 0.99–1.01) for the period from 1992 to 1996 and 0.90 (95% CI 0.86–0.93) for the period from 2017 to 2021 (Supplementary Figure 3J). The risk of rheumatoid arthritis in the birth cohort rose from 0.83 (95% CI 0.68–1.02) in 1902 to 1.19 (95% CI 0.93–1.54) in 2002 (Supplementary Figure 3J).

The incidence of gout in South Korea gradually increased with age, reaching its peak around 60 years old and then stabilizing. The incidence based on the period had been increasing year by year, [RRperiod (1992–1996) = 0.97, 95% CI 0.96–0.99], [RRperiod (2017–2021) = 1.06, 95% CI 1.04–1.07]. The risk of the birth cohort had increased from 1.05 (95% CI 0.95–1.15) in 1992 to 1.18 (95% CI 0.97–1.43) in 2002 (Supplementary Figure 3K). The incidence of low back pain increased significantly with age and reached its peak around 80 years old. The incidence based on the period had decreased year by year. The risk of the birth cohort had decreased from 1.11 (95% CI 1.04–1.19) in 1902 to 0.92 (95% CI 0.91–0.93) in 2002 (Supplementary Figure 3L). The incidence of neck pain increased significantly with age and reached its peak around 80 years old. The incidence based on the period had fluctuated slightly, and the risk of the birth cohort had slightly decreased in recent years (Supplementary Figure 3M). The incidence of osteoarthritis gradually increased with age and reached its peak around 70 years old before starting to decline. The incidence based on the period had increased year by year from 1992 to 2016 and gradually decreased from 2017 to 2021. The risk of the recent birth cohort had gradually increased (RRcohort 1992 = 0.97, 95% CI 1.01–1.14), (RRcohort 2002 = 1.19, 95% CI 1.12–1.27) (Supplementary Figure 3N). The incidence of rheumatoid arthritis gradually increased with age and reached its peak around 60 years old before gradually decreasing. The incidence based on the period had increased year by year from 1992 to 2016 and slightly decreased from 2017 to 2021. The risk of the recent birth cohort had gradually increased (RRcohort 1992 = 1.59, 95% CI 1.44–1.74), (RRcohort 2002 = 1.78, 95% CI 1.47–2.15) (Supplementary Figure 3).

### 3.4 Analysis of the temporal trend of risk factors

**Figure 4:**
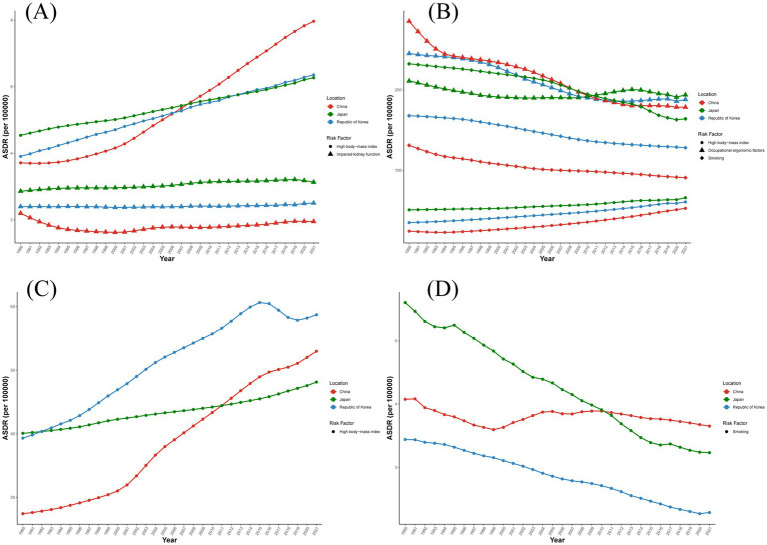
Contribution of major risk factors to ASDR of diseases from 1990 to 2021. **(A)** Contribution of high BMI and renal insufficiency to ASDR of gout. **(B)** Contribution of high BMI, smoking and occupational efficiency factors to ASDR of low back pain. **(C)** Contribution of high BMI to ASDR of osteoarthritis. **(D)** Contribution of smoking to ASDR of rheumatoid arthritis.

In the ASDR of gout, the increase in ASDR caused by high BMI in China rose from 3.5/100,000 in 1990 to 8/100,000 in 2021. In all three countries, the increase in ASDR caused by high BMI was relatively large, while the increase in ASDR caused by impaired renal function was relatively small (Figure 4A). In the ASDR caused by low back pain, the increase in ASDR caused by high BMI gradually rose and stabilized by 2021. However, the ASDR caused by occupational ergonomics factors and smoking was relatively high in 1990 and then gradually decreased and stabilized after 2000. In China and South Korea, the ASDR caused by occupational ergonomics factors was the highest, while in Japan, the ASDR caused by smoking was the highest (Figure 4B). In the ASDR of osteoarthritis, the increase in ASDR caused by high BMI showed an upward trend in all three countries, with the largest increase in South Korea. It rose from approximately 30/100,000 in 1990 to approximately 50/100,000 in 2021 (Figure 4C). In the ASDR of rheumatoid arthritis, the decrease in ASDR caused by smoking was a downward trend in all three countries, with a larger decrease in Japan and South Korea, from 5.61 (95% CI = 3.96–7.75) and 3.44 (95% CI = 2.40–4.68) per 100,000 people to 3.23 (95% CI = 2.12–4.71) and 2.28 (95% CI = 1.46–3.37) per 100,000 people (Figure 4D).

### 3.5 Forecasting ASIR for the next 15 years

**Figure 5:**
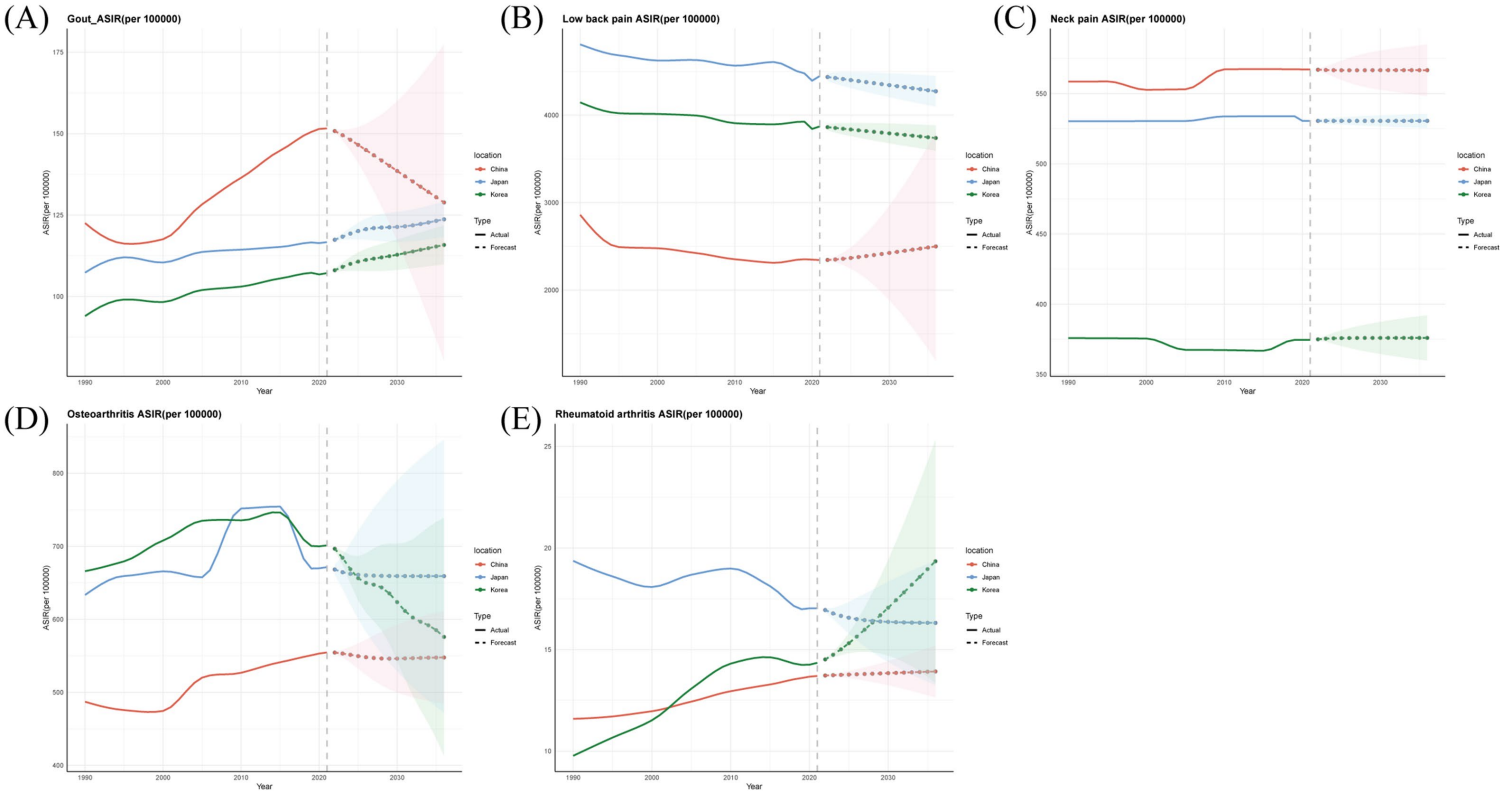
Predicted trends of five major musculoskeletal disorders ASIR in China, Japan and Republic of Korea over the next 15 years (2022–2036). The solid line represents the true trend of the ASIR rate from 1990 to 2021; the dashed lines and the shaded area represent the predicted trend and its 95% confidence interval. **(A)** Gout. **(B)** Low back pain. **(C)** Neck pain. **(D)** Osteoarthritis. **(E)** Rheumatoid arthritis.

The ARIMA model was used to quantitatively describe the trend of ASIR for the five major musculoskeletal diseases in China, Japan and South Korea over the next 15 years. It was predicted that the ASIR of gout in Japan and South Korea would continue to rise, while the ASIR of gout in China would decrease significantly from 151.6 per 100,000 people in 2021 to 128.9 per 100,000 people in 2036 (Figure 5A). It was predicted that the ASIR of low back pain in South Korea and Japan would continue to decline, while the ASIR of low back pain in China would increase from 2342.5/100,000 to 2498.4/100,000 (Figure 5B). It was predicted that the ASIR of neck pain would remain basically stable in all three countries (Figure 5C). It was predicted that the ASIR of osteoarthritis in China and Japan would slightly increase over the next 15 years, while the ASIR of osteoarthritis in South Korea would decrease from 701.2 per 100,000 people in 2021 to 575.9 per 100,000 people in 2036 (Figure 5D). It was predicted that the ASIR of rheumatoid arthritis would increase in both China and South Korea, with the ASIR in South Korea increasing from 14.3/100,000 in 2021 to 19.3/100,000 in 2036, while it would decrease from 17.0/100,000 to 16.3/100,000 in Japan (Figure 5E).

## 4 Discussion

### 4.1 Main findings

This research focuses on the incidence, prevalence, DALYs, ASIR, and ASDR of five musculoskeletal disorders—rheumatoid arthritis, osteoarthritis, low back pain, neck pain, and gout—across China, Japan, and South Korea between 1990 and 2021. These findings reveal a substantial rise in the prevalence of these conditions in 2021 relative to 1990 in all three nations. Notably, South Korea experienced the most dramatic surge in gout cases, with a 165.0% increase (95% UI 138.4–191.5%). Potential factors contributing to this significant rise included population growth and enhanced investments in healthcare infrastructure and workforce across these countries.

The results of the age-period-cohort analysis are consistent with previous epidemiological studies, indicating that the burden of disease caused by the five major musculoskeletal disorders increases with age in China, Japan, and South Korea, with a peak concentration between the ages of 40 and 80. As people age, the muscle mass in the body gradually decreases, and physical functions decline, leading to degeneration of joint cartilage and an increased susceptibility to musculoskeletal diseases (20). In addition, the probability of older adult suffering from chronic diseases is higher. Some chronic diseases, such as diabetes, can cause neuropathy and vascular diseases, affecting the blood supply and nerve regulation of muscles and bones. Therefore, population aging may be the main driving factor for the burden of musculoskeletal diseases. Moreover, China, Japan, and South Korea are typical countries with severe population aging. In recent years, China has adopted proactive measures to encourage childbirth, but the population growth effect remains unsatisfactory. According to statistics, the expected lifespan of 60-year-old males in China is 19.39 years, while that of females is 22.81 years (27). Therefore, the extension of life expectancy can offset the relatively small population growth caused by encouraging childbirth, and Japan and South Korea are already in a stage of negative population growth. In 2020, the fertility rate in South Korea dropped below 0.84, and the number of deaths reached 300,000 (28). The high degree of population aging has led to an increasingly heavy social and economic burden caused by musculoskeletal diseases. Patients require long-term treatment and care, and families and society need to invest a large amount of human, material, and financial resources, including medical expenses, nursing costs, rehabilitation costs, and indirect economic losses due to caring for patients. This undoubtedly brings a heavy burden to the society and economy. Therefore, the impact of musculoskeletal diseases on policies has become increasingly important. For example, in terms of improving medical care supply, the government has increased research investment and improved the diagnosis and treatment levels of diseases. At the same time, it continuously optimizes the allocation of medical resources and increases investment in grassroots medical institutions to improve the ability of grassroots medical institutions in the prevention, diagnosis, and treatment of musculoskeletal diseases. In addition, the retirement age can be extended to alleviate the burden of social older adult care and make full use of human resources. To a certain extent, it can alleviate the economic growth pressure caused by the reduction in the labor force and provide strong support for the stable development of the society and economy.

According to Joinpoint regression analysis, the ASIR and ASDR related to low back pain in the three countries have decreased over the past three decades, indicating that the disease has been well controlled. However, the ASIR and ASDR of gout, osteoarthritis, rheumatoid arthritis, and neck pain had all increased to varying degrees, represented that the control of the burden of these four diseases is still severe. Especially, the disease burden of osteoarthritis and gout had increased in all three countries, which required our greater attention and needed to increase investment in disease prevention and treatment. Osteoarthritis is a chronic progressive disease accompanied by irreversible structural changes. When the disease progresses to an advanced stage, it can seriously affect the quality of life of patients and cause a heavy social and economic burden. However, there is currently no clear diagnostic standard for early osteoarthritis, and no active management measures or sensitive efficacy evaluation methods can be adopted to test the intervention effect of early diseases (29). Therefore, this could be a contributing factor to the increasing disease burden of osteoarthritis in recent years. Gout is the only disease among the five major musculoskeletal diseases where the burden on males is higher than that on females. In 2021, the incidence of gout among males in China, Japan, and South Korea was 2.82, 2.53, and 2.91 times higher than that among females, respectively. Furthermore, the etiological factors of gout varied between the sexes. Females were at a higher risk of suffering from renal conditions and were often treated with thiazide diuretics or other agents that promote diuresis. In contrast, males were more likely to acknowledge a diet that is conducive to the occurrence and exacerbation of gout and were at a greater risk of developing hyperuricemia compared to females, a condition that precedes the onset of gout (12). Hypertension, the use of diuretics, genetic predisposition, excessive intake of alcohol, and a diet rich in purines have been frequently cited in earlier studies as contributing to the risk of gout (30–32). These elements heightened the susceptibility to gout.

The AAPC of the ASIR and ASDR for neck pain changed little over the past three decades, suggesting that the efforts to alleviate its disease burden differ from those for low back pain and had not yet produced satisfactory results. A potential explanation could be that shifts in lifestyle and the implementation of successful preventive measures during recent decades contributed to a decline in the prevalence of low back pain (33). However, due to the insufficient attention paid by researchers, clinicians, and the entire society to neck pain, the majority of the working-age population spent most of their time in office environments, resulting in an increase in the burden of neck pain. This indicates that policies needed to be formulated to prevent such occupational hazards (34). In both China and South Korea, the ASIR for rheumatoid arthritis increased while the ASDR decreased, suggesting that despite the rising incidence of rheumatoid arthritis with socioeconomic development, the burden of disability caused by the disease is decreasing due to effective pharmaceutical management. As an autoimmune disease, the incidence of rheumatoid arthritis was gradually increasing globally, and it was higher in developed regions than in less developed regions (17). In Japan, ASIR gradually decreased, indicating that Japan is performing better than the global average in addressing the burden of rheumatoid arthritis.

In the context of China, Japan, and South Korea, the detrimental effects of smoking on rheumatoid arthritis and low back pain, the role of workplace ergonomic factors in low back pain, and the consequences of reduced kidney function on gout incidence all demonstrated a declining pattern. Conversely, the association between high BMI and conditions such as osteoarthritis, low back pain, and gout showed a persistent upward trajectory. This phenomenon may be attributed to age-related systemic fat deposition and the growing population of older adults with elevated BMI levels. The aging process tended to make individuals less active, leading to a reduction in energy expenditure, which in turn affected energy metabolism and subsequently increased the risk of obesity (35). Genetic epidemiological studies utilizing Mendelian randomization approaches established a significant causal link between BMI and low back pain and chronic pain syndromes (36). Research by Lee et al. (37) highlighted that increased body mass exerted compressive forces on lumbar vertebral structures and intervertebral discs, predisposing to low back pain. Beyond joint loading, excessive mechanical stress accelerates cartilage degradation, initiating osteoarthritic changes (38, 39). Furthermore, elevated BMI contributes to gout pathogenesis through increased serum urate concentrations (40), promoting monosodium urate crystal deposition and systemic inflammatory responses (41). Consequently, monitoring BMI in older adult populations, promoting appropriate physical activity, and enhancing metabolic efficiency may mitigate the impact of musculoskeletal disorders. In areas experiencing increasing disease burdens related to high BMI, implementing evidence-based weight control strategies, improving nutritional profiles, and encouraging regular physical activity could help manage individual BMI levels and curb disease progression. Ultimately, implementing population-level interventions targeting BMI reduction may alleviate the burden of major musculoskeletal conditions.

Enhancing public participation in physical activities is an important component for achieving the goals of the “Healthy China 2030” planning outline (42). Therefore, the Chinese government has successively released the “Physical Activity Guidelines for Chinese People (2021) (43) and A Guide for the Prevention and Treatment of Work-related Musculoskeletal Disorders (2022), providing scientific and detailed exercise guidance for different groups of people, aiming to control body weight, reduce the national BMI, enhance muscle strength, and reduce the incidence of musculoskeletal diseases. In the health policy “Healthy Japan 21 (Second term)” in the 21st century (44), Japan shifted its focus from simply prolonging life expectancy to a disease-free life and improving quality of life, and then focused on achieving the ideal health status of each individual. The main action goals of this plan include increasing the average daily step count of the public and increasing the number of adults with regular exercise habits by approximately 10% within 10 years. The South Korean government has also formulated multiple health promotion plans, such as the “5th National Comprehensive Health Enhancement Plan (HP2030),” aiming to increase the national healthy life expectancy, promote health equity, and expand the coverage of health. South Korea has also implemented the “Happy Healthy Village” project (45), aiming to improve the health level of residents by improving their lifestyle habits (such as controlling obesity, quitting smoking, and improving diet). Although China, Japan, and South Korea have taken various policies and measures to reduce BMI and promote physical activity among the public and have achieved certain results, the solution to the obesity problem still requires long-term policy support and full participation of the public.

Monitoring and predicting disease trends are critical components of disease prevention and control. Based on the predictions from the ARIMA model, it was projected that by 2036, the health burden of the top five musculoskeletal disorders in China, Japan, and South Korea will remain substantial. The incidence rates of low back pain in China, gout in Japan and rheumatoid arthritis in South Korea were predicted to increase from 2021 to 2036. However, at present, the attention paid by public health institutions and the public to musculoskeletal diseases was still insufficient. The huge difference between high incidence rates and low treatment rates may have to some extent explained the reason why the DALYs of musculoskeletal diseases remained high. Therefore, we propose the development of legal documents such as a “Health Promotion Law” to reform the medical system and establish a three-tiered prevention system. This system includes preventing risk factors at the primary care level, disseminating relevant knowledge to the older adult and high-risk populations, conducting early screening, and providing high-quality medical services to reduce the burden of musculoskeletal diseases and improve patient health outcomes.

### 4.2 Research intensity

This research presents the most extensive assessment of the incidence rates of the five major musculoskeletal disorders in China, Japan, and South Korea, with an emphasis on their temporal patterns, and demonstrated the critical role of targeted investments in diseases and risk factors for the prevention and management of such disorders. The GBD study employed consistent and standardized analytical methods, facilitating the comparison of these estimates across different time periods. The temporal patterns may represent the most crucial aspect in understanding the disease burden from these musculoskeletal conditions in the three countries. Moreover, the study not only examined the overall changes over the entire period (assessed by AAPC) but also analyzed each distinct time segment (assessed by APC) using the Joinpoint regression analysis. Ultimately, forecasting the disease burden aids in providing vital evidence for policy development and interventions aimed at specific conditions, and supports governments in setting priorities. By investing in public health initiatives for musculoskeletal disorders early on, governments can mitigate the severity of these conditions at the population level, particularly focusing on reducing the preventable burden among the youth.

### 4.3 Research limitations

This study has several limitations that must be acknowledged. Firstly, while the GBD 2021 made adjustments to data sources to account for the variability across different studies and to standardize data, these modifications inherently introduced greater uncertainty into the analyzed data. Secondly, the actual national data on the annual incidence and point prevalence of the five musculoskeletal disorders is derived from a limited number of countries. The findings presented here are largely based on the DisMod-MR 2.1 (2, 11) modeling process. Consequently, the interpretation of temporal trend estimates should be approached with caution. Promoting the inclusion of musculoskeletal conditions in national health data gathering and incorporating these data into models is essential for ensuring the acquisition of more comprehensive and precise information. Thirdly, the absence of provincial-level data for each country in our study precludes a detailed geographical representation of the burden of these major musculoskeletal diseases. Lastly, the age-period-cohort analysis provides population-level insights but may be subject to ecological bias.

## 5 Conclusion

China, Japan, and South Korea are faced with significant burdens due to musculoskeletal disorders. There is a pressing need to elevate public awareness regarding the risk factors, impacts, and evidence-based therapeutic approaches for these conditions. Special attention should be paid to osteoarthritis among older adult females and gout among older adult males, which have caused significant disease burdens. In addition, the impact of high BMI on gout, low back pain and osteoarthritis is continuously increasing. It is suggested that the government increase investment, optimize resource allocation, and carry out prevention and control work such as weight management and health education.

## Data Availability

Publicly available datasets were analyzed in this study. This data can be found at: https://vizhub.healthdata.org/gbd-results/.

## Footnotes

^1^ https://vizhub.healthdata.org/gbd-results/
