# Supplementary material for: Burden, trends, and predictions of five major musculoskeletal disorders in China, Japan, and South Korea: analysis based on the Global Burden of Disease Study 2021
Source: Front Public Health. 2025 Jun 13;13:1582618. doi: 10.3389/fpubh.2025.1582618 (PMC12202420; doi:10.3389/fpubh.2025.1582618)
Supplement: Supplementary file 1 [file Data_Sheet_1.docx]

Supplementary Figure 1.


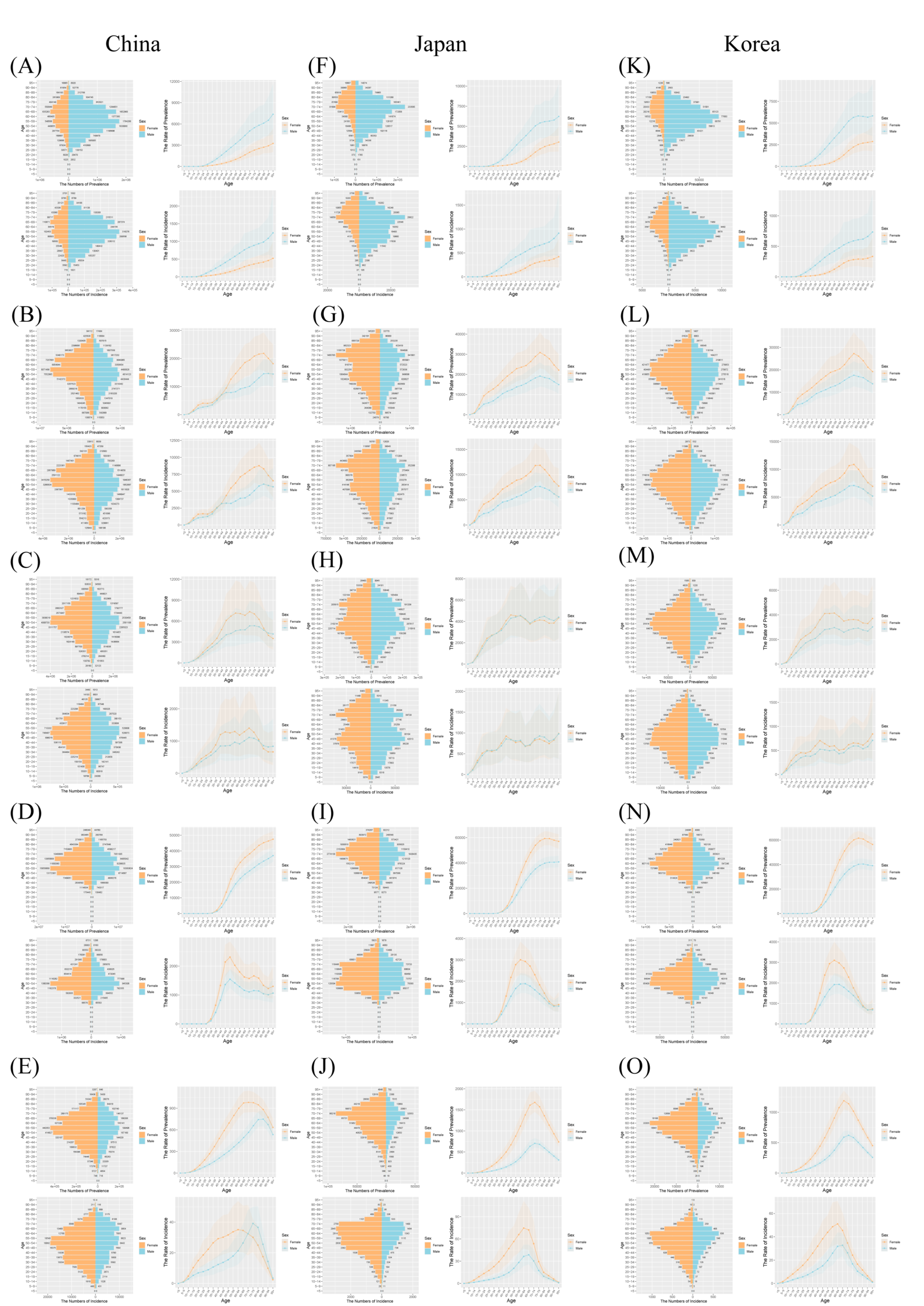


The incidence rates, the number of cases, the prevalence rates, and the number of cases of the five major musculoskeletal diseases in China, Japan and South Korea. Gout (A, F, K), Low back pain (B, G, L), Neck pain (C, H, M), Osteoarthritis (D, I, N), Rheumatoid arthritis (E, J, O).

Supplementary Figure 2.


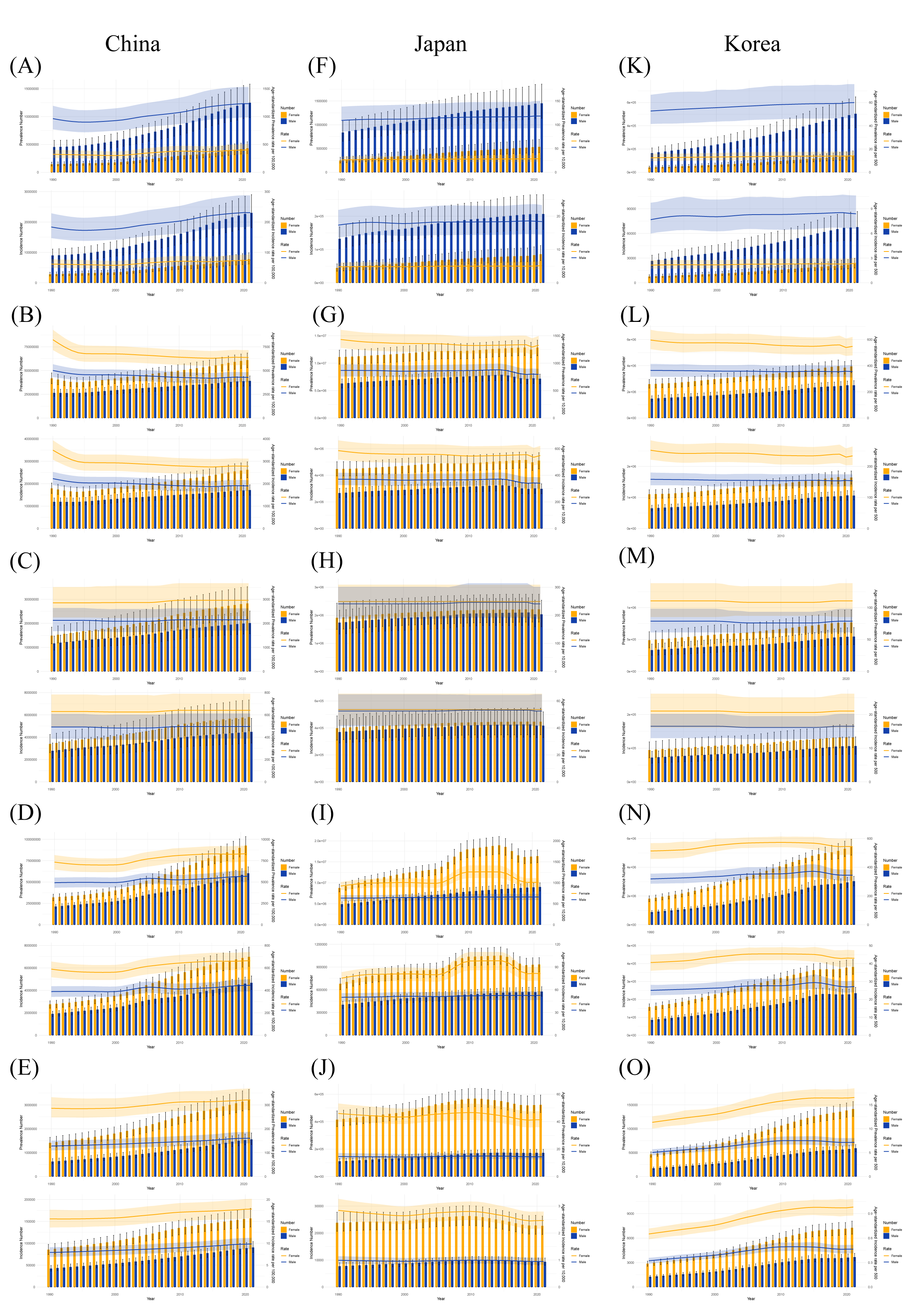


The ASIR (age-standardized incidence rate), the number of cases and ASDR (age-standardized disability rate), and the number of patients with the five major musculoskeletal diseases in China, Japan and South Korea. Gout (A, F, K), Low back pain (B, G, L), Neck pain (C, H, M), Osteoarthritis (D, I, N), Rheumatoid arthritis (E, J, O).

Supplementary Figure 3.


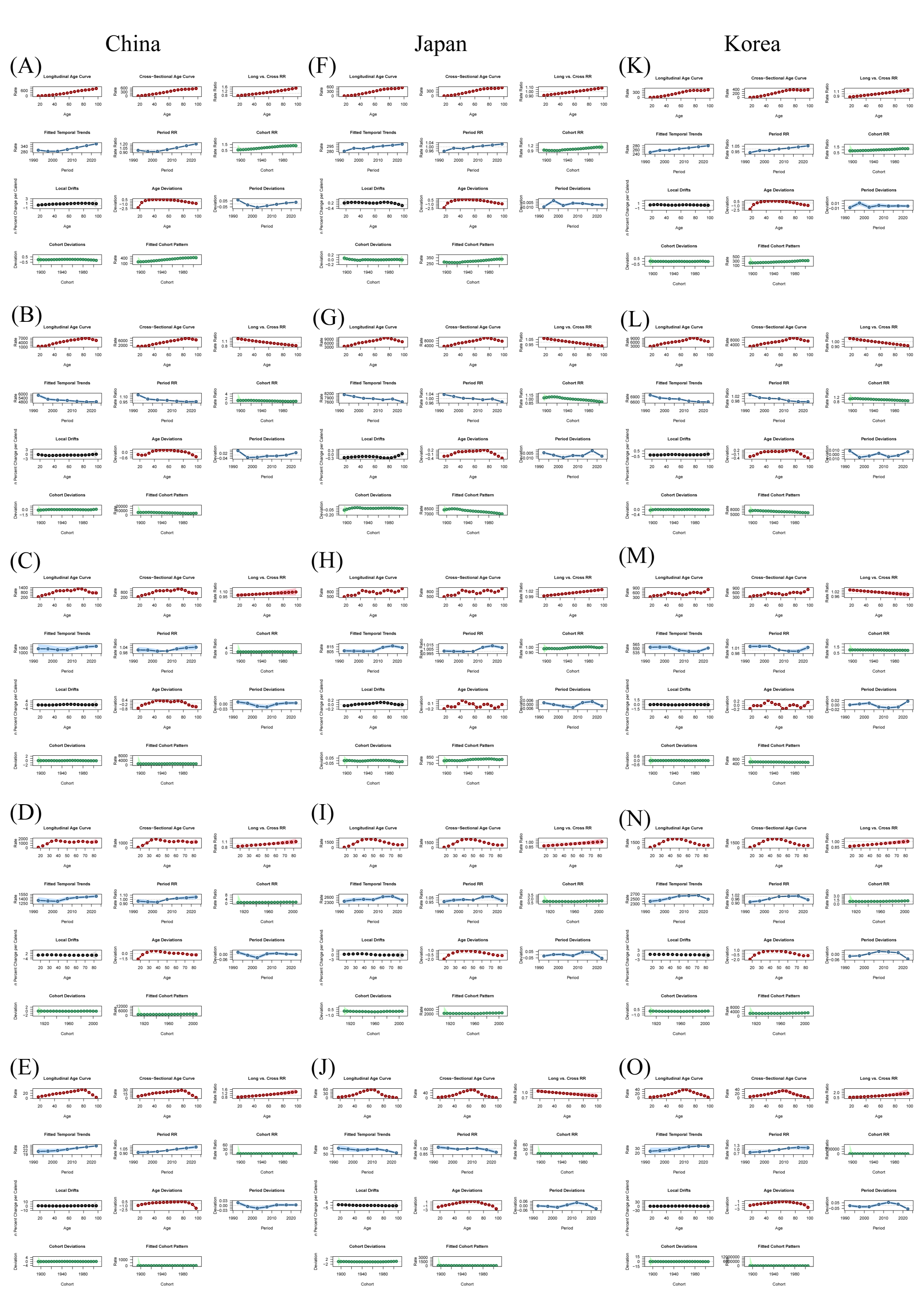


The age-period-cohort analysis of incidence rates of five major musculoskeletal diseases in China, Japan and South Korea. Gout (A, F, K), Low back pain (B, G, L), Neck pain (C, H, M), Osteoarthritis (D, I, N), Rheumatoid arthritis (E, J, O).
